# DNA methylation of HPA-axis genes and the onset of major depressive disorder in adolescent girls: a prospective analysis

**DOI:** 10.1038/s41398-019-0582-7

**Published:** 2019-10-03

**Authors:** Kathryn L. Humphreys, Sarah R. Moore, Elena Goetz Davis, Julie L. MacIsaac, David T. S. Lin, Michael S. Kobor, Ian H. Gotlib

**Affiliations:** 1 Department of Psychology and Human Development, Vanderbilt University, Nashville, TN USA; 2 BC Children’s Hospital Research Institute, University of British Columbia, Vancouver, BC Canada; 3 Department of Psychology, Stanford University, Stanford, USA

**Keywords:** Depression, Clinical genetics

## Abstract

The stress response system is disrupted in individuals with major depressive disorder (MDD) as well as in those at elevated risk for developing MDD. We examined whether DNA methylation (DNAm) levels of CpG sites within HPA-axis genes predict the onset of MDD. Seventy-seven girls, approximately half (*n* = 37) of whom were at familial risk for MDD, were followed longitudinally. Saliva samples were taken in adolescence (M age = 13.06 years [SD = 1.52]) when participants had no current or past MDD diagnosis. Diagnostic interviews were administered approximately every 18 months until the first onset of MDD or early adulthood (M age of last follow-up = 19.23 years [SD = 2.69]). We quantified DNAm in saliva samples using the Illumina EPIC chip and examined CpG sites within six key HPA-axis genes (*NR3C1*, *NR3C2*, *CRH*, *CRHR1*, *CRHR2*, *FKBP5*) alongside 59 genotypes for tagging SNPs capturing cis genetic variability. DNAm levels within CpG sites in *NR3C1*, *CRH, CRHR1*, and *CRHR2* were associated with risk for MDD across adolescence and young adulthood. To rule out the possibility that findings were merely due to the contribution of genetic variability, we re-analyzed the data controlling for cis genetic variation within these candidate genes. Importantly, methylation levels in these CpG sites continued to significantly predict the onset of MDD, suggesting that variation in the epigenome, independent of proximal genetic variants, prospectively predicts the onset of MDD. These findings suggest that variation in the HPA axis at the level of the methylome may predict the development of MDD.

## Introduction

Major depressive disorder (MDD) is among the most prevalent of all psychiatric disorders, affecting ~9% of adult women each year^1^. MDD is also characterized by high recurrence rates and functional impairment^2,3^, resulting in enormous personal and societal costs and burden^4^. In order to identify targets that can be used in approaches to prevent and/or treat MDD, investigators have worked to elucidate factors involved in the etiology of this disorder. In this context, research in behavioral genetics has documented high heritability estimates for MDD, suggesting that a large proportion of risk for depression is attributable to genetic factors^5^. Indeed, consistent with this formulation, children of depressed parents are at significantly higher risk for developing MDD than are offspring of non-affected parents^6^. Authors who reported on the largest genome-wide association study of MDD concluded that the disorder results from a “complex process of intertwined genetic and environmental effects^7^.” Indeed, children of depressed parents not only shared increased genetic risks for this disorder, but also experienced environmental factors that may increase their risk for developing MDD, including exposure to stress hormones in utero^8^ and adverse caregiving environments during childhood^9,10^. The intersection of these environmental and genetic risk factors can be captured, at least in part, by epigenetic markers^11–13^; in fact, it has been suggested that epigenetic markers will prove to be stronger predictors of the development of depression than will genes alone^14^.

Multiple studies have documented gene by environment interactions in the prediction of depression and provide a compelling backdrop against which to examine epigenetic factors that influence the development of MDD^14^. In particular, researchers have posited that common polymorphisms in genes involved in hypothalamic–pituitary–adrenal (HPA)-axis functioning (i.e., genes for the glucocorticoid and mineralocorticoid receptors (*NR3C1* and *NR3C2*, respectively), the corticotropin-releasing hormone (*CRH*), CRH receptors type 1 and type 2 (*CRHR1* and *CRHR2*), and the FK506-binding protein (*FKBP5*)), contribute to variability in stress reactivity to increase risk for MDD^15–18^. In the HPA-axis stress cascade, corticotropin-releasing factor (CRF) receptors 1 and 2 are primarily involved in the initiation and the termination of the stress response, respectively^19^, with *CRHR2* having lower affinity for CRF^20^. Glucocorticoid receptor activation through high concentrations of corticosteroids functions as part of the negative feedback process within the HPA axis via actions on gene transcription^21^. Genetic variation in *CRHR1* has been associated with depression following childhood maltreatment^22^, with a blunted cortisol response to stress^23^, and with the severity of psychotic depression^16^. *CRHR2* has been associated in particular with stress sensitivity and anxious behavior^24^, although associations with psychiatric phenotypes have been weaker than is the case for *CRHR1*, possibly due to its role in modulating the *CRHR1*-mediated stress response^25^. Genetic variation in *NR3C1* has been implicated both in the pathophysiology of depression^17^ and in elevated cortisol and psychotic symptoms in MDD^16^; in addition, DNA methylation (DNAm) at this gene has been linked to stressful life experiences and stress reactivity^26^.

DNAm is an epigenetic modification to the DNA sequence corresponding with gene expression, with the specific nature of the relation depending on the genomic context^27^. DNAm of loci within *NR3C1* and *FKBP5* have previously been linked to alterations in the functioning of the HPA-axis-mediated stress response^28–33^. Moreover, DNAm is responsive to environmental input^28,31,34^ and has been shown to interact with genetic variation to predict brain and behavioral outcomes^35,36^; thus, DNAm of HPA-axis genes may be an important signature of the biological impact of environmental exposures on the stress response system. Given that this system is disrupted not only in individuals with MDD^37^, but also in individuals who are at elevated risk for developing MDD^38^, DNAm of loci within HPA axis genes may be informative in identifying whom may go on to develop MDD.

In this longitudinal study of girls at familial risk for depression, we examined whether DNAm within specific HPA-axis genes can predict the subsequent onset of MDD. We recruited a sample of 9- to 14-year-old girls who themselves had no past or present diagnosis of MDD but who either were or were not at familial risk for depression^39–41^. We assessed and followed the girls prospectively into adulthood, or until they experienced their first depressive episode. Although some recent research has found no methylation sites that survived correction in relation to MDD^42^, several prior studies reported concurrent depressive symptoms and diagnoses were associated with DNAm using various approaches^43–47^. Given the biological relevance of HPA-axis function to risk for MDD, and that epigenetic variation may reflect combined genetic and environmental risks, we hypothesized that DNAm in CpG sites in HPA-axis genes assessed in early adolescence would predict the onset of MDD. In addition, given the theoretical and empirical work linking non-inherited environmental factors to MDD^14,48^, we hypothesized that DNAm levels would predict the onset of MDD over and above both common genetic variants in these HPA-axis genes and familial risk for MDD.

## Materials and methods

Participants were recruited on the basis of being at high or low familial risk for depression by virtue of their mothers’ history of MDD. Specifically, mothers either had experienced at least two episodes of MDD during their daughter’s life or had no current or past diagnosis of MDD. For this study, daughters were included in the analyses only if they had no current or past MDD at the time the saliva sample was obtained for DNAm quantification. The 77 girls in this sample (Mage saliva sample = 13.06 years, SD = 1.52) included 37 at familial risk for MDD and 40 without familial risk; for this study of DNAm, these two subgroups of girls were selected both to be comparable in the age at which they gave their saliva sample and to include approximately similar cell sizes for a 2 × 2 design (familial risk; developed MDD). The majority of the girls reported their race/ethnicity as Caucasian (65%), followed by multiracial (8%), Asian (8%), Hispanic (6%), “Other” (6%), and African American (5%). Participants and their mothers completed diagnostic assessments about the daughter at regular intervals (approximately every 18 months) to determine the age of the daughter’s first onset of MDD^49,50^. All procedures were approved by the Stanford Institutional Review Board, and assent and informed consent were obtained from the participants and their parents, respectively.

### Psychiatric interview

MDD was assessed using the Kiddie Schedule for Affective Disorders^51^; both the participants and their mothers provided reports regarding the participants’ mental health symptoms at the initial assessment. We conducted follow-up structured interviews approximately every 18 months, to assess whether daughters had developed MDD within the most recent interval (see ref. ^50^). When a participant met full diagnostic criteria for MDD, the age of onset of the first episode of MDD was ascertained and follow-up assessments ceased. Participants who endorsed no current or past MDD episodes were followed until early adulthood or we were unable to contact them.

### DNA sample processing and DNAm microarray

Saliva samples were collected using Oragene kits (DNA Genotek, Inc., Ottawa, ON, Canada) and genomic DNA was extracted using the DNeasy Kit (Qiagen, Hilden, Germany). DNA samples were genotyped for 59 tagging single-nucleotide polymorphisms (SNPs) across *NR3C1*, *NR3C2*, *CRH*, *CRHR1*, *CRHR2*, and *FKBP5* using iPlex reagents on a MassArray System (Agena Bioscience, Inc., San Diego, CA, USA), using standard conditions according to the manufacturer’s protocol, or using a Taqman platform from Applied Biosystems (for SNP selection, see ref. ^16^ and Supplementary Table 1 for a list of SNPs and gene locations). Genotypes were verified to be in Hardy–Weinberg equilibrium and were coded as the number of minor alleles (0,1,2). DNAm assaying has been described previously^52^. Briefly, the EZ DNA Methylation Kit (Zymo Research, Irvine, CA, USA) provided bisulfite-converted DNA for whole-genome amplification and enzyme fragmentation, then hybridized to a MethylationEPIC BeadChip (Illumina, San Diego, CA, USA), a genome-wide platform that assesses over 850,000 CpG sites. Processed BeadChips were scanned on an Illumina HiScan and intensity values were imported into GenomeStudio (Illumina, San Diego, CA, USA) for data quality assessment, color correction, and background subtraction. A data matrix was then exported as *β*-values representing percent DNAm ranging from 0 to 1 (0 = unmethylated, 1 = methylated). As described previously^53^, DNAm data were preprocessed to remove SNP probes, polymorphic probes, cross-hybridizing probes^54^, probes located on the X and Y chromosomes, and probes with missing values or detection *p*-values > 0.01. DNAm data were then normalized, corrected for technical batch effects and for the proportion of cell types (a mixture of buccal epithelial cells, and CD34 leukocytes were found to vary in saliva samples) using computational deconvolution^55^. Within the CpG sites in the six HPA-axis genes outlined above, we reduced our tested sites to variable CpGs (Table 1 indicates the number of variable CpGs tested per gene), subsetting to probes in which the variability of *β*-values between the 10th and 90th percentile was > 5%.

**Table 1:** Summary of the CpGs, SNPs, and SNP PCs tested

Gene	Number of variable CpGs	Number of SNPs	Number of PCs	Hits (no SNP) models	Hits (+SNP) models
*NR3C1*	39	8	4	3	4
*NR3C2*	18	12	3	-	-
*FKBP5*	26	1	-	-	-
*CRH*	11	5	3	1	1
*CRHR1*	42	17	2	2	2
*CRHR2*	16	16	5	1	3

Hits based on a nominal *p*-value threshold (*p* *<* 0.01)

### Data analysis

All analyses used the full sample of participants (*N* = 77). First, we determined whether there were significant correlations between cis genetic variants and DNAm. As our goal was to control for genetic variability rather than to identify possible biological effects of SNPs, we applied principal component analysis to reduce the set of SNPs for each gene to a reduced number of uncorrelated components to use as covariates. This strategy maximally accounts for genetic variability and avoids using multiple correlated covariates in models.

Second, we examined DNAm as a predictor of MDD onset using Cox proportional hazards regression analysis, a type of survival model ideal for use with a quantitative predictor (i.e., DNAm). Survival analysis accounts for the time-to-event (age of MDD onset, in years) design. Participants were categorized as having developed MDD or not, and the time-to-event variable was the age of MDD onset or the age at the last assessment at which no current or past episode of MDD was reported. In the first round, we assessed in independent models which CpGs across candidate genes significantly predicted MDD, controlling for familial risk, ethnicity, and age. In the second round, we included as covariates principal components (PCs) generated from SNPs within each gene. As multiple test correction using false discovery rate (FDR) is overly conservative when multiple CpG sites are highly correlated^56^, we applied a more liberal FDR threshold of <0.2.

## Results

The associations begween cis genetic variants and DNAm are presented in Supplementary Fig. 1a-f. Multiple SNPs within each gene were significantly intercorrelated and were also moderately associated with CpG sites. Table 1 presents the CpGs, SNPs, and the number of PCs and hits identified in models (both without cis genetic variants and including SNP PC covariates) for each candidate gene.

Of the 77 participants in this study, 33 (43%) met the criteria for MDD over the follow-up period. Although we attempted to obtain a roughly equal distribution of girls who did and did not go on to develop MDD based on familial MDD risk, a greater proportion of girls at familial risk for depression (60%) developed MDD than did girls at low familial risk (29%; *χ*^2^(1) = 8.02, *p* = 0.005). As noted above, we included risk status as a covariate in our statistical models. Overall, onset of MDD occurred at a mean age of 15.45 years (SD = 1.56, range 11–18). High- and low-risk girls who developed MDD did not differ significantly in age of onset of MDD (M[SD] = 15.23[1.69] vs. 15.91[1.22] years; *t*(31) = 1.19, *p* = 0.244). Girls who did not develop MDD during the follow-up period were last assessed at age 19.23[2.69] years.

In our first round of models, in which genetic PCs were not examined, we identified seven CpG sites that significantly predicted onset of MDD at FDR adjusted *p* < 0.2, corresponding to a nominal *p* < 0.01, in *NR3C1*, *CRH*, *CRHR1*, and *CRHR2*. In a second set of analyses, in which we covaried for genetic variation using PCs, we found that these same CpG sites remained significant in the prediction of MDD. In addition, these analyses with genetic variation covaried identified three additional CpG sites in the same genes, indicating that six CpG sites significantly predicted the onset of MDD above and beyond the genetic variability. Supplementary Tables 2 and 3 show results for all models tested. The direction and approximate magnitudes of significant effect estimates were consistent between models in which we controlled for and did not control for proximal genetic effects.

**Fig. 1:**
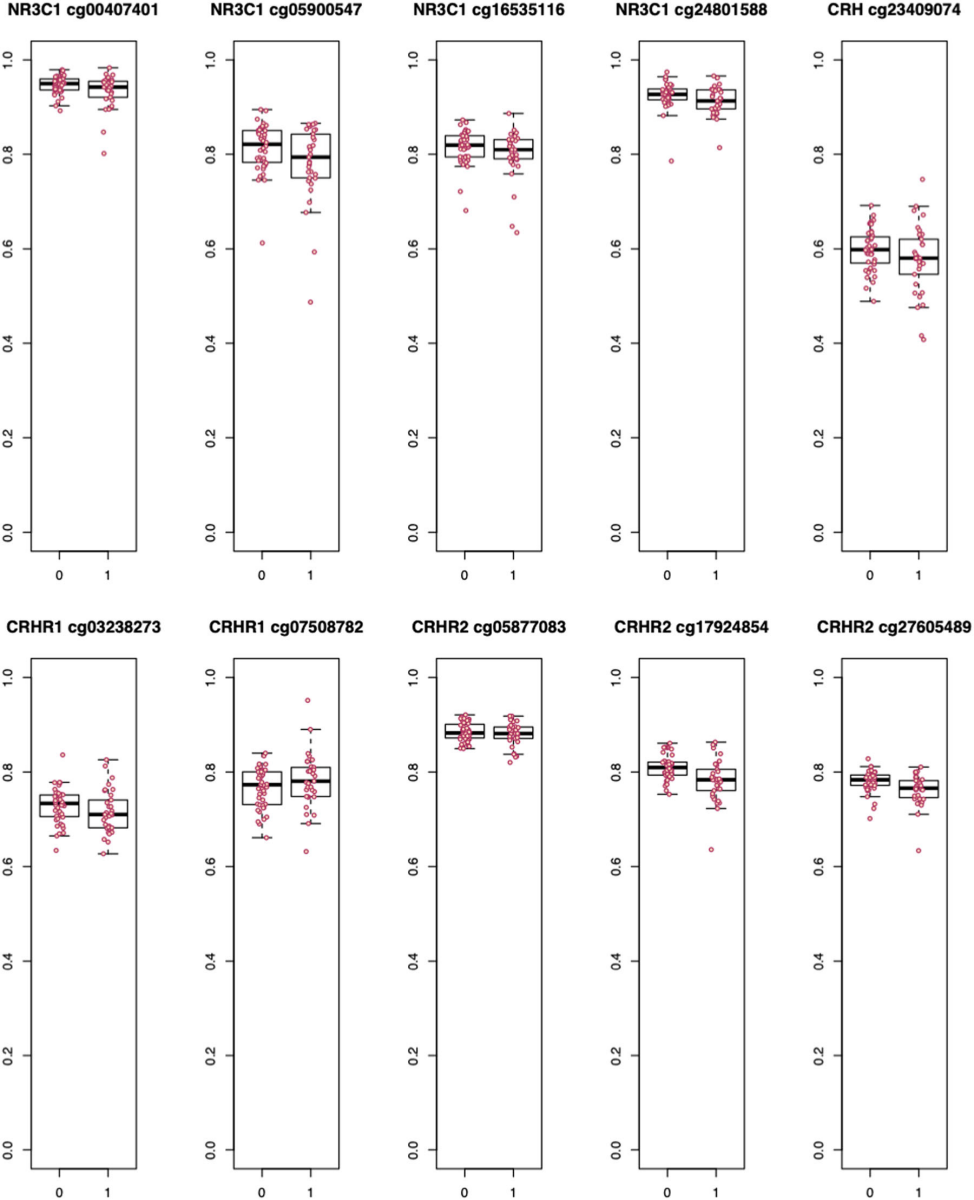
Differences in DNAm *β*-values by depression onset for all significant CpGs. 0 = No MDD; 1 = MDD onset

**Fig. 2:**
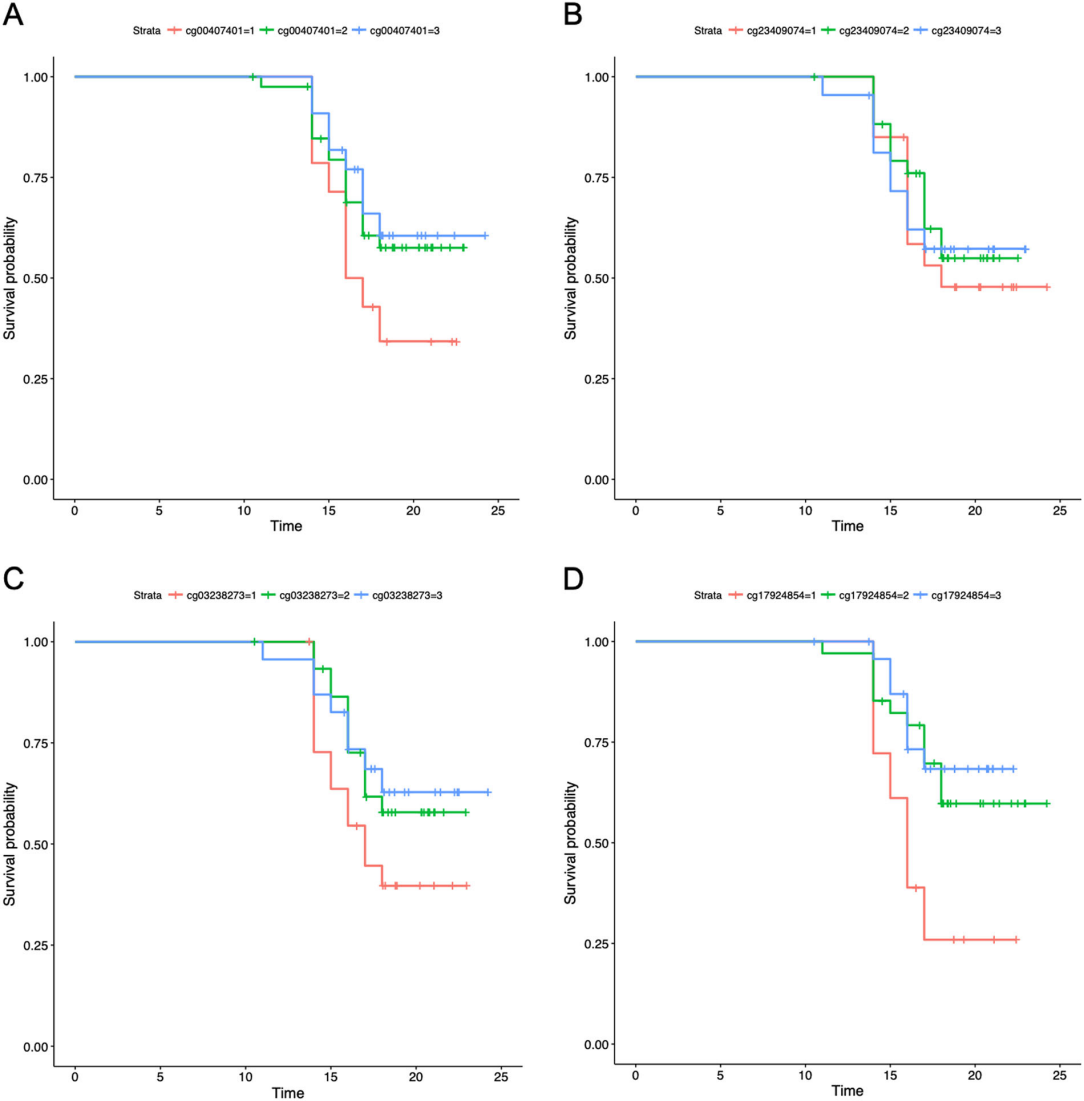
**a**–**d** Example survival plots from each candidate gene with significant hits. DNAm *β*-values binned by ± 0.5 SD from the mean (1 = below average, 2 = average, and 3 = above average). In these four examples, survival probability (of no MDD onset) is lower with hypomethylation. **a** *NR3C1*. **b** *CRH*. **c** *CRHR1*. **d** *CRHR2*

To visualize the raw effect sizes of DNAm in association with depression, we plot the raw DNAm *β*-values for individuals with and without MDD for each significant CpG in Fig. 1. As shown, in all but one site, DNAm is negatively associated with the onset of depression. The raw differences in *β*-values or “Delta Betas” are presented in Table 2, as are Cohen’s *d* calculations. Although these Delta Betas are considered small in the broader epigenetics literature, small effects have been validated in large cohorts and have been shown to have meaningful downstream associations with gene expression^57^. Example survival curves binned by DNAm levels, which visualize the data with regards to the full model (i.e., adjusting for covariates), are presented in Fig. 2a–d. In these examples, the curves show that “survival” probability (of no MDD onset) is lower when DNAm levels are below average and higher when DNAm levels are above average. In other words, these results suggest that lower levels of methylation are associated with greater odds of MDD onset.

**Table 2:** Effect sizes

	Raw Delta *β*	Cohen’s *d*	*p*-value	Adjusted *p*-value
NR3C1 cg00407401	−0.014	−0.495	0.00	0.11
NR3C1 cg05900547	−0.032	−0.486	0.00	0.11
NR3C1 cg16535116	−0.015	−0.335	0.01	0.13
NR3C1 cg24801588	−0.011	−0.386	0.00	0.09
CRH cg23409074	−0.020	−0.347	0.01	0.13
CRHR1 cg03238273	−0.013	−0.325	0.01	0.13
CRHR1 cg07508782	0.016	0.332	0.01	0.13
CRHR2 cg05877083	−0.005	−0.207	0.01	0.15
CRHR2 cg17924854	−0.027	−0.788	0.01	0.13
CRHR2 cg27605489	−0.018	−0.636	0.00	0.03

*N* = 77. Raw delta *β* = average difference in DNAm *β*-values between depressed and nondepressed groups, not controlling for covariates. Cohen’s *d* is an effect size or standardized difference between groups

In Table 3 we present the functional annotation information for all significant CpGs. In *NR3C1*, all four CpGs are located in the gene body, with two sites located in proximity to a transcription start site (TSS), one site associated with an enhancer element, one falling in a region of open chromatin, and two sites located within a transcription factor-binding site (TFBS) region. The *CRH* CpG is located on a CpG island shore in close proximity to a TSS and within a TFBS region. The two sites in *CRHR1* are within an untranslated region, with one falling in both a region of open chromatin and TFBS region. Finally, the three sites in *CRHR2* are all located within the gene body. Of these three sites, one also falls within a CpG island shore and is in proximity to a TSS, and a second is associated with an enhancer element.

**Table 3:** Functional annotation information for all CpG hits

	Gene	Chromosome	Mapinfo	Strand	UCSC RefGene Group	CpG Island	X450k Enhancer	GencodeBasicV12 Group	OpenChromatin	TFBS
cg00407401	*NR3C1*	5	142690959	R	Body			3′-UTR	chr5:142690878–142691553	chr5:142690757–142691933
cg05900547	*NR3C1*	5	142769791	F	Body			TSS1500;5′-UTR		
cg16535116	*NR3C1*	5	142769612	R	Body		True	TSS200;5′-UTR		chr5:142761726–142769708
cg24801588	*NR3C1*	5	142689858	F	Body			3′-UTR		
cg23409074	*CRH*	8	67090798	F	TSS200	S Shore		5′-UTR;1stExon		chr8:67088813–67091549
cg03238273	*CRHR1*	17	43825672	F	5′-UTR			5′-UTR;5′-UTR		
cg07508782	*CRHR1*	17	43783158	R	5′-UTR			5′-UTR;5′-UTR	chr17:43782663–43783355	chr17:43782647–43783225
cg05877083	*CRHR2*	7	30703457	F	Body					
cg17924854	*CRHR2*	7	30706895	R	Body		True			
cg27605489	*CRHR2*	7	30723603	F	TSS1500;Body	S Shore		TSS1500		

*450 k Enhancer* predicted enhancer elements as annotated in the original 450 K design; *Genecode Basic V12 Group* gene region feature category describing the CpG position from basic Genecode; *Mapinfo* chromosomal coordinates of the CpG (Build 37); *Open Chromatin* chromosomal coordinates of open chromatin region from ENCODE; *S* downstream (3′) of CpG island*TFBS* chromosomal coordinates of transcription factor binding site region from ENCODE; *TSS* transcription start site; *UCSC RefGene Group* gene region feature category describing the CpG position from UCSC; *UTR* untranslated region

A negative relation with depression = lower DNAm in depression cases

## Discussion

In the present study, we examined whether DNAm levels at loci within six genes that have been implicated in HPA-axis functioning (i.e., *FKBP5*, *NR3C1*, *NR3C2*, *CRH*, *CRHR1*, and *CRHR2*) prospectively predict the onset of MDD in a sample of 77 girls at low and high familial risk for MDD, who had no past or current MDD diagnosis at baseline. We found that DNAm levels within CpG sites in *NR3C1*, *CRH*, *CRHR1*, and *CRHR2* were associated with risk for MDD across adolescence and young adulthood. Further, as variation in common SNPs from these genes may be responsible, at least in part, for these associations, we re-analyzed the data controlling for genetic variation and for familial risk for MDD. Importantly, methylation levels continued to be a significant predictor of the onset of MDD and additional CpG sites were identified as significantly associated with MDD, suggesting that non-inherited factors (e.g., environmental factors) predict the onset of MDD through variation in levels of DNAm over and above the effects of genetic variation and familial risk. These data indicate that there are likely both genetic and epigenetic contributions to depression risk, but because of the modifiability of DNAm, epigenetic markers may be a more useful independent predictor of MDD risk.

Researchers have taken diverse approaches in examining patterns of DNAm that are associated with major depression; perhaps, not surprisingly, a wide range of findings have been reported. Most studies have found that the the CpG sites (using candidate and epigenome-wide approaches) associated with concurrent depressive symptoms or diagnosis^43–47^ were characterized by lower levels of methylation (c.f., ref. ^58^). It should be noted, however, that the number and selection of CpG sites, the tissue assayed, and the covariates examined have varied considerably, possibly leading to inconsistent findings of the sites identified. Moreover, as methodological approaches improve to ensure rigorous testing of DNAm hypotheses (e.g., importance of considering the large contribution of cell type to DNAm patterns^59^), focusing on theoretically motivated sites of interest is critical for both practical (i.e., reducing Type I and II errors) and programmatic (i.e., identifying possible targets for intervention) reasons^60^. A theoretically targeted approach that is based on specific genes or regions yields a tighter range of CpG sites that should be examined and simultaneously allows for greater specificity. We followed this approach of condensing a large search space by selecting, *a priori*, genes that have been linked to HPA-axis functioning and vulnerability to depression^16^, and by focusing only on variable sites. It is also critical to note that previous studies have examined the association between DNAm and concurrent depression. Given that DNAm levels have been found to be associated concurrently with psychopathology^61^, our approach of quantifying DNAm prior to the onset of MDD and prospectively assessing its predictive association with depression advances our understanding of the temporal relation between DNAm and depression. In previous research, investigators have predicted depression in the postpartum period from antenatal DNAm (focusing on loci at CpGs cg21326881 and cg00058938, in the promoter regions of the *HP1BP3* and *TTC9B* genes, respectively)^62^; in that study, however, the sample included a large percentage of women with a history of mood disorders, thereby reducing confidence in elucidating the directional nature of this association.

In addition to the advantage in the present study of being able to examine temporal directionality of DNAm associations, it is noteworthy that the majority of significant sites are located in proximity to functional elements in the genome, suggesting that our findings reflect meaningful biological differences in the regulation of the stress response. For instance, two sites fall within regions of an open chromatin; thus, given the accessibility of DNA to regulatory elements, they are likely to link to patterns of gene expression. In addition, multiple sites are in proximity to TSSs and enhancers, where DNAm levels may have implications for regulatory binding. Not surprisingly, given our focus on CpG sites characterized by sufficient variability in DNAm levels, we found all of our sites to fall within gene bodies or untranslated regions; no sites were within gene promoters. Gene promoters are most often unmethylated across an entire population of cells and, therefore, are so invariable that it is difficult to distinguish any small effect from the technicial variation of DNAm assays^59^. Typically, DNAm in gene promoters is negatively associated with gene expression and positively associated in gene bodies^27^. These assocations, however, are not straightforward and depend on tissue type, development, and nuances of genomic context (e.g., the abundance of particular transcription factors)^63^. In the present study we found associations within salivary DNA; these cells are only indirectly relevant to downstream effects of the central stress response. Nevertheless, even if DNAm is simply epiphenominal and variations appear downstream of a functional change and in other tissues, they may still be informative of biological history relevant to the etiology of MDD.

We should note five limitations of this study. First, we measured DNAm in saliva. Although some researchers have suggested that saliva and buccal epithelial cell samples are more relevant than blood samples in terms of concordance with brain tissue^55,64^, saliva is not the primary tissue of interest for central regulation of the stress response. Moreover, saliva is one of the most accessible tissues for human (epi)genomics and our results provide preliminary evidence that patterns of DNAm in saliva are a biological predictor for the risk of MDD onset. Second, although the prospective design of this study allows us to examine predictors of MDD onset, it does not allow us to make mechanistic determinations; instead, these findings should be interpreted as informing our understanding of the etiology of MDD. Third, the present sample included only girls and only 77 individuals; replicating these findings in larger samples that also include males would increase our confidence that DNAm in these candidate gene loci predicts MDD and would confirm whether these findings are specific to females. Fourth, we used a targeted approach to examine DNAm levels. By focusing on CpG sites within six genes that function in the HPA axis, we used theoretically motivated selections (i.e., based on previous research linking MDD with the stress response system^16,18,49,50^) to develop an empirically informed hypothesis and narrow the search space. Although we controlled for common SNPs within these genes, other genetic variants across the genome may have trans effects on the DNAm sites we examined. Of course, other genes of interest are associated with MDD^7,65^, it is also important to consider other disorders (e.g., anxiety and eating disorders) as relevant outcomes. Our decision to follow participants only until they experienced their first depressive episode prevents us from being able to examine the development of other forms of psychopathology; moreover, given our growing understanding of potential latent factors that underlie many forms of psychopathology^66^, the links among genetic risk, methylation, and psychiatric symptoms are broader than we could capture here. Finally, we did not assess environmental contributors to the observed DNAm levels. It will be important in future research to examine potential environmental factors, pathways to levels of DNAm, and epigenome by environment interactions^67^.

Despite these limitations, this study is important in documenting that variation in DNAm levels within key genes of the HPA axis prospectively predict the onset of MDD. By demonstrating that epigenetic variation potentially linked to HPA-axis functioning is altered prior to the emergence of MDD in a manner that predicts the onset of MDD, the present study extends previous research linking anomalous HPA-axis functioning to depression. It will be important in future research to replicate these findings in a larger sample and to determine whether epigenetic variation in these markers are linked to biological byproducts of HPA-axis activity, including cortisol production both diurnally and in response to stress. Further, prospective assessments (i.e., repeated methylation assessments within individuals) paired with careful assessments of potential epigenetic modifiers (e.g., stressful life experiences; endogenous hormone production during puberty) and broad-based assessments of functioning would facilitate the detection of the timing, potential causes, and consequences of epigenetic changes in HPA axis and other genes. Identifying these potentially malleable risk factors for depression can inform the generation of targets that can be used to measure the impact of intervention and prevention efforts on biological factors that may mediate the relation between risk for depression and the onset of MDD.

## Supplementary information

Supplemental Material

## References

[1] Substance Use and Mental Health Administration. Key substance use and mental health indicators in the United States: results from the 2016 National Survey on Drug Use and Health. In: 2*016 Natl Surv Drug Use Heal* 2017. https://www.samhsa.gov/data/sites/default/files/NSDUH-FFR1-2016/NSDUH-FFR1-2016.htm.

[2] Richards D (2011). Prevalence and clinical course of depression: a review. Clin. Psychol. Rev..

[3] Kessler RC, McGonagle KA, Swartz M, Blazer DG, Nelson CB (1993). Sex and depression in the National Comorbidity Survey I: lifetime prevalence, chronicity and recurrence. J. Affect Disord..

[4] Greenberg PE, Fournier A-A, Sisitsky T, Pike CT, Kessler RC (2015). The economic burden of adults with major depressive disorder in the United States (2005 and 2010). J. Clin. Psychiatry.

[5] Sullivan PF, Neale MC, Kendler KS (2000). Genetic epidemiology of major depression: review and meta-analysis. Am. J. Psychiatry.

[6] Gotlib, I. H. & Colich, N. L. in *Handbook of Depression* (eds Gotlib, I. H. & Hammen C.L.) p. 240–258 (Guilford Press, New York, 2014).

[7] Wray N. R. et al. Genome-wide association analyses identify 44 risk variants and refine the genetic architecture of major depression. *Nat. Genet.* **50**, 668–681 (2018). 10.1038/s41588-018-0090-3. 10.1038/s41588-018-0090-3 PMC5934326 29700475

[8] Field T (2008). Prenatal dysthymia versus major depression effects on maternal cortisol and fetal growth. Depress. Anxiety.

[9] Field T (2010). Postpartum depression effects on early interactions, parenting, and safety practices: a review. Infant Behav. Dev..

[10] Kendler KS, Gardner CO (2017). Genetic and environmental influences on last-year major depression in adulthood: a highly heritable stable liability but strong environmental effects on 1-year prevalence. Psychol. Med..

[11] Meaney MJ (2010). Epigenetics and the biology of gene x environment interactions. Child Dev..

[12] Boyce W. T., Kobor M. S. Development and the epigenome: The ‘synapse’ of gene-environment interplay. *Dev. Sci.* **18**, 1–23 (2015). 10.1111/desc.12282. 10.1111/desc.12282 25546559

[13] Halldorsdottir T., Binder E. B. Gene × Environment Interactions: From Molecular Mechanisms to Behavior. *Annu. Rev. Psychol*. **68**, 215–241 (2017). 10.1146/annurev-psych-010416-044053. 10.1146/annurev-psych-010416-044053 27732803

[14] Heim C, Binder EB (2012). Current research trends in early life stress and depression: review of human studies on sensitive periods, gene-environment interactions, and epigenetics. Exp. Neurol..

[15] Davis E. G. et al. Corticotropin-releasing factor 1 receptor haplotype and cognitive features of major depression. *Transl. Psychiatry* **8**, 5 (2018). 10.1038/s41398-017-0051-0. 10.1038/s41398-017-0051-0 PMC5802461 29317606

[16] Schatzberg AF (2014). HPA axis genetic variation, cortisol and psychosis in major depression. Mol. Psychiatry.

[17] Van Rossum EFC (2006). Polymorphisms of the glucocorticoid receptor gene and major depression. Biol. Psychiatry.

[18] Papiol S (2007). Genetic variability at HPA axis in major depression and clinical response to antidepressant treatment. J. Affect Disord..

[19] De Kloet ER, Joëls M, Holsboer F (2005). Stress and the brain: from adaptation to disease. Nat. Rev. Neurosci..

[20] Lewis K (2001). Identification of urocortin III, an additional member of the corticotropin-releasing factor (CRF) family with high affinity for the CRF2 receptor. Proc. Natl Acad. Sci. USA.

[21] Derijk RH (2009). Single nucleotide polymorphisms related to HPA axis reactivity. Neuroimmunomodulation.

[22] Polanczyk G (2009). Protective effect of CRHR1 gene variants on the development of adult depression following childhood maltreatment: replication and extension. Arch. Gen. Psychiatry.

[23] Tyrka AR (2009). Interaction of crhildhood maltreatment with the corticotropin-releasing hormone receptor gene: effects on hypothalamic-pituitary-adrenal axis reactivity. Biol. Psychiatry.

[24] Bale TL (2000). Mice deficient for corticotropin-releasing hormone receptor-2 display anxiety-like behaviour and are hypersensitive to stress. Nat. Genet..

[25] Binder EB, Nemeroff CB (2010). The CRF system, stress, depression and anxiety: Insights from human genetic studies. Mol. Psychiatry.

[26] Palma-Gudiel H, Córdova-Palomera A, Leza JC, Fañanás L (2015). Glucocorticoid receptor gene (NR3C1) methylation processes as mediators of early adversity in stress-related disorders causality: a critical review. Neurosci. Biobehav. Rev..

[27] Jones M. J., Moore S. R., Kobor M. S. Principles and challenges of applying epigenetic epidemiology to psychology. *Annu. Rev. Psychol.* **69**, 459–485 (2018). 10.1146/annurev-psych-122414-033653 29035689

[28] Oberlander TF (2008). Prenatal exposure to maternal depression, neonatal methylation of human glucocorticoid receptor gene (NR3C1) and infant cortisol stress responses. Epigenetics.

[29] Argentieri MA, Nagarajan S, Seddighzadeh B, Baccarelli AA, Shields AE (2017). Epigenetic pathways in human disease: the impact of DNA methylation on stress-related pathogenesis and current challenges in biomarker development. EBioMedicine.

[30] Meaney MJ, Szyf M (2005). Environmental programming of stress responses through DNA methylation: life at the interface between a dynamic environment and a fixed genome. Dialogues Clin. Neurosci..

[31] Unternaehrer E., Meinlschmidt G. Psychosocial Stress and DNA Methylation. *Epigenet. Neuroendocrinol. Clin. Focus Psychiatry* **2**, 227–261 (2016).

[32] Matosin N, Halldorsdottir T, Binder EB (2018). Understanding the molecular mechanisms underpinning gene by environment interactions in psychiatric disorders: the FKBP5 model. Biol. Psychiatry.

[33] Turecki G, Meaney MJ (2016). Effects of the social environment and stress on glucocorticoid receptor gene methylation: a systematic review. Biol. Psychiatry.

[34] Non A. L. et al. DNA methylation at stress‐related genes is associated with exposure to early life institutionalization. *Am. J. Phys. Anthropol*. **16**, 84–93 (2016). 10.1002/ajpa.23010 PMC5278953 27218411

[35] Jaenisch R, Bird A (2003). Epigenetic regulation of gene expression: how the genome integrates intrinsic and environmental signals. Nat. Genet..

[36] Ursini G (2011). Stress-related methylation of the catechol-O-methyltransferase Val158 allele predicts human prefrontal cognition and activity. J. Neurosci..

[37] Pariante CM, Lightman SL (2008). The HPA axis in major depression: classical theories and new developments. Trends Neurosci..

[38] LeMoult J, Chen MC, Foland-Ross LC, Burley HW, Gotlib IH (2015). Concordance of mother-daughter diurnal cortisol production: understanding the intergenerational transmission of risk for depression. Biol. Psychol..

[39] Foland-Ross LC, Gilbert BL, Joormann J, Gotlib IH (2015). Neural markers of familial risk for depression: an investigation of cortical thickness abnormalities in healthy adolescent daughters of mothers with recurrent depression. J. Abnorm. Psychol..

[40] Gotlib IH (2010). Neural processing of reward and loss in girls at risk for major depression. Arch. Gen. Psychiatry.

[41] Waugh CE, Muhtadie L, Thompson RJ, Joormann J, Gotlib IH (2012). Affective and physiological responses to stress in girls at elevated risk for depression. Dev. Psychopathol..

[42] Bustamante AC, Armstrong DL, Uddin M (2018). Epigenetic profiles associated with major depression in the human brain. Psychiatry Res..

[43] Boström AE (2017). A MIR4646 associated methylation locus is hypomethylated in adolescent depression. J. Affect Disord..

[44] Khulan B. et al. Epigenomic profiling of men exposed to early-life stress reveals DNA methylation differences in association with current mental state. *Transl. Psychiatry* **4**, e448 (2014). 10.1038/tp.2014.94. 10.1038/tp.2014.94 PMC4203020 25247593

[45] Dempster EL (2014). Genome-wide methylomic analysis of monozygotic twins discordant for adolescent depression. Biol. Psychiatry.

[46] Kaut O (2015). Aberrant NMDA receptor DNA methylation detected by epigenome-wide analysis of hippocampus and prefrontal cortex in major depression. Eur. Arch. Psychiatry Clin. Neurosci..

[47] Weder N. et al. Child abuse, depression, and methylation in genes involved with stress, neural plasticity, and brain circuitry. *J.**Am. Acad. Child Adolesc. Psychiatry* **53**, 417–424e.5 (2014). 10.1016/j.jaac.2013.12.025. 10.1016/j.jaac.2013.12.025 PMC4126411 24655651

[48] Adrian C, Hammen C (1993). Stress exposure and stress generation in children of depressed mothers. J. Consult Clin. Psychol..

[49] Colich NL, Kircanski K, Foland-Ross LC, Gotlib IH (2015). HPA-axis reactivity interacts with stage of pubertal development to predict the onset of depression. Psychoneuroendocrinology.

[50] LeMoult J, Ordaz SJ, Kircanski K, Singh MK, Gotlib IH (2015). Predicting first onset of depression in young girls: Interaction of diurnal cortisol and negative life events. J. Abnorm. Psychol..

[51] Kaufman J., Birmaher B., Brent D., Rao U., Ryan N. Kiddie-Sads-Present and Lifetime Version (K-SADS-PL). http://www.psychiatry.pitt.edu/node/8233. 1996.

[52] Davis EG (2017). Accelerated DNA methylation age in adolescent girls: associations with elevated diurnal cortisol and reduced hippocampal volume. Transl. Psychiatry.

[53] Moore SR (2017). Epigenetic correlates of neonatal contact in humans. Dev. Psychopathol..

[54] Zhou W, Laird PW, Shen H (2017). Comprehensive characterization, annotation and innovative use of Infinium DNA methylation BeadChip probes. Nucleic Acids Res.

[55] Smith A. K. et al. DNA extracted from saliva for methylation studies of psychiatric traits: evidence tissue specificity and relatedness to brain. *Am. J. Med. Genet. B Neuropsychiatr. Genet*. **168B**, 36–44 (2015). 10.1002/ajmg.b.32278. 10.1002/ajmg.b.32278 PMC4610814 25355443

[56] Zhou Z (2019). Correction for multiple testing in candidate-gene methylation studies. Epigenomics.

[57] Breton C. V. et al. Small-magnitude effect sizes in epigenetic end points are important in children’s environmental health studies: the children’s environmental health and disease prevention research center’s epigenetics working group. *Environ. Health Perspect*. **125**, 511–526 (2017). 10.1289/EHP595. 10.1289/EHP595 PMC5382002 28362264

[58] Roy B., Shelton R. C., Dwivedi Y. DNA methylation and expression of stress related genes in PBMC of MDD patients with and without serious suicidal ideation. *J.**Psychiatr. Res.* **89**, 115–124 (2017). 10.1016/j.jpsychires.2017.02.005. 10.1016/j.jpsychires.2017.02.005 PMC5391149 28246044

[59] Moore S. R., Kobor M. S. Variability in DNA methylation at the serotonin transporter gene promoter: epigenetic mechanism or cell-type artifact? *Mol. Psychiatry* (2018). 10.1038/s41380-018-0121-6. [Epub ahead of print]. 10.1038/s41380-018-0121-6 PMC7473835 30082839

[60] McDade T. W. et al. Social and physical environments early in development predict DNA methylation of inflammatory genes in young adulthood. *Proc. Natl Acad. Sci. USA* **114**, 7611–7616 (2017). 10.1073/pnas.1620661114. 10.1073/pnas.1620661114 PMC5530653 28673994

[61] Braithwaite EC, Kundakovic M, Ramchandani PG, Murphy SE, Champagne FA (2015). Maternal prenatal depressive symptoms predict infant NR3C1 1F and BDNF IV DNA methylation. Epigenetics.

[62] Guintivano J, Arad M, Gould TD, Payne JL, Kaminsky ZA (2014). Antenatal prediction of postpartum depression with blood DNA methylation biomarkers. Mol. Psychiatry.

[63] Maurano MT (2015). Role of DNA methylation in modulating transcription factor occupancy. Cell Rep..

[64] Lowe R (2013). Buccals are likely to be a more informative surrogate tissue than blood for epigenome-wide association studies. Epigenetics.

[65] Lohoff FW (2010). Overview of the genetics of major depressive disorder. Curr. Psychiatry Rep..

[66] Caspi A., Moffitt T. E. All for one and one for all: mental disorders in one dimension. *Am. J. Psychiatry* **175**, 831–844 (2018). 10.1176/appi.ajp.2018.17121383. 10.1176/appi.ajp.2018.17121383 PMC6120790 29621902

[67] Radtke K. M. et al. Epigenetic modifications of the glucocorticoid receptor gene are associated with the vulnerability to psychopathology in childhood maltreatment. *Transl. Psychiatry* **5**, e571 (2015). 10.1038/tp.2015.63. 10.1038/tp.2015.63 PMC4471294 26080088

