# Supplementary material for: DNA methylation of HPA-axis genes and the onset of major depressive disorder in adolescent girls: a prospective analysis
Source: Transl Psychiatry. 2019 Oct 3;9:245. doi: 10.1038/s41398-019-0582-7 (PMC6776528; doi:10.1038/s41398-019-0582-7)
Supplement: Supplementary file 1 — Supplemental Material [file 41398_2019_582_MOESM1_ESM.docx]

Supplementary material

Supplemental Table 1. rs number for each single nucleotide polymorphism (SNP) and gene, location of the SNP

| SNP rs number | Gene and location of SNP |
| --- | --- |
| rs1360780 | FKBP5 Intron |
| rs6198 | NR3C1 3' UTR |
| rs33388 | NR3C1 Intron |
| rs2918419 | NR3C1 Intron |
| rs10052957 | NR3C1 Intron |
| rs10482633 | NR3C1 Intron |
| rs12521436 | NR3C1 Promoter |
| rs17209258 | NR3C1 Intron |
| rs41423247 | NR3C1 Intron |
| rs5525 | NR3C2 Coding exon D‎/D |
| rs1879829 | NR3C2 Intron (boundary) |
| rs2070951 | NR3C2 5' UTR |
| rs3910052 | NR3C2 Intron |
| rs4835488 | NR3C2 Intron |
| rs6535578 | NR3C2 Intron |
| rs7658048 | NR3C2 Intron |
| rs7694064 | NR3C2 Intron |
| rs10213471 | NR3C2 Promoter |
| rs17484245 | NR3C2 Intron |
| rs2070950 | NR3C2 Intron |
| rs5522 | NR3C2 Coding exon V‎/I |
| rs10098823 | CRH Promoter |
| rs3176921 | CRH Promoter |
| rs5030875 | CRH Promoter |
| rs7350113 | CRH Downstream |
| rs110402 | CRH Intron - TAT tag |
| rs16940674 | CRHR1 coding exon C‎/TER |
| rs171440 | CRHR1 Intron |
| rs17689966 | CRHR1 Intron |
| rs242924 | CRHR1 Intron - TAT |
| rs242940 | CRHR1 Intron |
| rs242948 | CRHR1 3' UTR |
| rs4076452 | CRHR1 Intron |
| rs4792887 | CRHR1 Intron |
| rs7209436 | CRHR1 Intron - TAT |
| rs4792825 | CRHR1 Intron |
| rs4458044 | CRHR1 Intron |
| rs12944712 | CRHR1 Intron |
| rs17763104 | CRHR1 Intron |
| rs2664008 | CRHR1 Intron |
| rs17763658 | CRHR1 Downstream |
| rs242942 | CRHR1 Intron |
| rs11657992 | CRHR1 Downstream |
| rs2240403 | CRHR2 Coding Exon S/S |
| rs2267712 | CRHR2 Intron |
| rs2267717 | CRHR2 Intron |
| rs2270007 | CRHR2 Intron |
| rs4723003 | CRHR2 Promoter |
| rs7812133 | CRHR2 Intron (boundary) |
| rs255102 | CRHR2 Promoter |
| rs975537 | CRHR2 Intron |
| rs2190242 | CRHR2 Intron |
| rs2267716 | CRHR2 Intron |
| rs2284216 | CRHR2 Intron |
| rs2284217 | CRHR2 Intron |
| rs4723000 | CRHR2 Intron (boundary) |
| rs12701020 | CRHR2 Intron |
| rs17159371 | CRHR2 Promoter |
| rs929377 | CRHR2 Intron |

Supplemental Table 2. All tested model results before adding SNP PCs

| CpG | Gene | Coefficient | Exponent | se | z | P Value | Adjusted P Value |
| --- | --- | --- | --- | --- | --- | --- | --- |
| cg27605489 | CRHR2 | -19.355603 | 3.93E-09 | 5.36124354 | -3.6102823 | 0.00030586 | 0.0271537 |
| cg24801588 | GR1 | -7.5908808 | 0.00050504 | 2.12643006 | -3.5697768 | 0.00035729 | 0.0271537 |
| cg00407401 | GR1 | -16.267913 | 8.61E-08 | 4.89103053 | -3.3260707 | 0.0008808 | 0.04462699 |
| cg05900547 | CRH | -8.7532777 | 0.00015794 | 3.06918773 | -2.8519851 | 0.00434471 | 0.16509912 |
| cg07508782 | GR1 | -9.7038751 | 6.10E-05 | 3.50196432 | -2.7709806 | 0.00558878 | 0.16989879 |
| cg03238273 | CRHR1 | 9.19679091 | 9865.41915 | 3.44001605 | 2.67347326 | 0.00750703 | 0.17769334 |
| cg16535116 | CRHR1 | -12.414799 | 4.06E-06 | 4.69472574 | -2.6444142 | 0.00818325 | 0.17769334 |
| cg23409074 | CRHR2 | -11.116578 | 1.49E-05 | 4.44791969 | -2.4992758 | 0.01244474 | 0.23645007 |
| cg17924854 | GR1 | -13.860329 | 9.56E-07 | 5.81199121 | -2.3847815 | 0.01708927 | 0.28861872 |
| cg05877083 | CRHR2 | -13.146805 | 1.95E-06 | 5.69763152 | -2.3074159 | 0.02103165 | 0.31968103 |
| cg05087823 | CRHR1 | -11.983471 | 6.25E-06 | 5.55359838 | -2.157785 | 0.03094455 | 0.42759746 |
| cg06952416 | FK | 5.28586192 | 197.524359 | 2.54815038 | 2.07439167 | 0.03804295 | 0.45710937 |
| cg06087101 | CRHR1 | -12.333002 | 4.40E-06 | 5.97764661 | -2.0631868 | 0.03909488 | 0.45710937 |
| cg08215831 | GR2 | -6.064596 | 0.0023237 | 2.99711797 | -2.0234759 | 0.0430241 | 0.46711885 |
| cg01972879 | CRH | -6.2400409 | 0.00194978 | 3.23180347 | -1.9308231 | 0.05350493 | 0.4786852 |
| cg24353392 | CRH | -10.888282 | 1.87E-05 | 5.68144795 | -1.9164625 | 0.05530625 | 0.4786852 |
| cg18640030 | CRHR1 | -13.427549 | 1.47E-06 | 7.1666356 | -1.8736197 | 0.06098287 | 0.4786852 |
| cg25719092 | GR2 | -8.477297 | 0.00020814 | 4.55105465 | -1.8627104 | 0.06250302 | 0.4786852 |
| cg04227637 | GR2 | -8.8547498 | 0.0001427 | 4.84912525 | -1.826051 | 0.06784259 | 0.4786852 |
| cg22046703 | CRH | -9.6161395 | 6.66E-05 | 5.2887457 | -1.8182269 | 0.06902947 | 0.4786852 |
| cg27225476 | FK | -9.1645206 | 0.00010469 | 5.04083581 | -1.8180558 | 0.06905561 | 0.4786852 |
| cg19650300 | GR2 | -7.2936092 | 0.00067987 | 4.04595965 | -1.8026896 | 0.07143698 | 0.4786852 |
| cg03591753 | CRHR2 | 15.0433619 | 3413886.45 | 8.37422166 | 1.79638927 | 0.07243263 | 0.4786852 |
| cg06843189 | FK | -5.9848579 | 0.00251657 | 3.41371405 | -1.7531808 | 0.07957098 | 0.50394955 |
| cg04856689 | GR1 | -7.454648 | 0.00057875 | 4.37756784 | -1.70292 | 0.08858303 | 0.53363167 |
| cg05437692 | CRHR1 | -11.22856 | 1.33E-05 | 6.64927776 | -1.6886887 | 0.0912791 | 0.53363167 |
| cg09238384 | GR1 | -12.121915 | 5.44E-06 | 7.29554323 | -1.6615507 | 0.0966029 | 0.54383857 |
| cg23416081 | GR1 | 12.7464011 | 343314.116 | 7.87768459 | 1.61803903 | 0.10565419 | 0.56856114 |
| cg01294526 | CRHR1 | -6.9518809 | 0.00095683 | 4.34562728 | -1.5997416 | 0.10965591 | 0.56856114 |
| cg18998365 | FK | -7.8533583 | 0.00038845 | 4.98797886 | -1.574457 | 0.11538183 | 0.56856114 |
| cg03857453 | GR1 | -5.2664459 | 0.00516192 | 3.4141746 | -1.5425239 | 0.12294633 | 0.56856114 |
| cg06475362 | CRHR2 | -13.577384 | 1.27E-06 | 8.84739609 | -1.5346193 | 0.12487738 | 0.56856114 |
| cg03066966 | CRHR2 | -8.0166622 | 0.00032992 | 5.22582809 | -1.5340463 | 0.12501826 | 0.56856114 |
| cg06175988 | GR1 | -4.2479733 | 0.01429317 | 2.81087673 | -1.5112628 | 0.13072152 | 0.56856114 |
| cg16664570 | GR2 | 8.46507244 | 4746.07121 | 5.65946662 | 1.49573679 | 0.13472226 | 0.56856114 |
| cg10993059 | GR2 | -7.9841701 | 0.00034082 | 5.35865101 | -1.489959 | 0.13623503 | 0.56856114 |
| cg19014730 | CRHR2 | -6.4643137 | 0.00155806 | 4.41000861 | -1.4658279 | 0.14269518 | 0.56856114 |
| cg17342132 | GR1 | -5.1164247 | 0.00599743 | 3.51557674 | -1.4553586 | 0.14557002 | 0.56856114 |
| cg20598211 | CRH | -6.0536003 | 0.00234939 | 4.16273515 | -1.4542362 | 0.14588082 | 0.56856114 |
| cg20728768 | FK | -6.9527425 | 0.00095601 | 4.87908823 | -1.4250086 | 0.15415473 | 0.58578796 |
| cg25708981 | FK | -8.0367448 | 0.00032336 | 5.69409754 | -1.4114168 | 0.15812176 | 0.5862075 |
| cg24063856 | GR1 | -6.0387634 | 0.00238451 | 4.45585979 | -1.3552409 | 0.17534082 | 0.59769437 |
| cg19226017 | FK | 2.94954788 | 19.0973175 | 2.19707066 | 1.34249114 | 0.1794368 | 0.59769437 |
| cg21789597 | CRH | -8.9094025 | 0.00013511 | 6.6587032 | -1.3380087 | 0.18089362 | 0.59769437 |
| cg03906910 | GR1 | 6.94947559 | 1042.60283 | 5.23774655 | 1.32680639 | 0.18457274 | 0.59769437 |
| cg17578833 | CRHR1 | -4.3482375 | 0.01292958 | 3.33536206 | -1.3036778 | 0.19234346 | 0.59769437 |
| cg16219186 | CRHR1 | 5.64729957 | 283.524791 | 4.35425553 | 1.29696099 | 0.19464461 | 0.59769437 |
| cg11760414 | CRHR2 | 5.58558925 | 266.557305 | 4.36896926 | 1.27846842 | 0.20108431 | 0.59769437 |
| cg23776787 | GR1 | 6.52098937 | 679.25008 | 5.14324847 | 1.26787368 | 0.20484307 | 0.59769437 |
| cg21517946 | FK | -6.9964336 | 0.00091514 | 5.53398602 | -1.2642666 | 0.20613436 | 0.59769437 |
| cg07817266 | CRHR1 | -9.3866226 | 8.38E-05 | 7.57230407 | -1.2395993 | 0.21512365 | 0.59769437 |
| cg19645279 | GR1 | 6.77458629 | 875.317161 | 5.53932666 | 1.22299816 | 0.22133039 | 0.59769437 |
| cg06613263 | FK | -4.1879994 | 0.01517662 | 3.433231 | -1.219842 | 0.22252477 | 0.59769437 |
| cg18484679 | GR2 | -5.8881568 | 0.00277208 | 4.89980549 | -1.2017124 | 0.22947499 | 0.59769437 |
| cg01819552 | CRHR1 | 4.71876271 | 112.029554 | 3.97825407 | 1.1861391 | 0.23556737 | 0.59769437 |
| cg12888360 | CRHR1 | -8.3668343 | 0.00023245 | 7.05495799 | -1.185951 | 0.23564166 | 0.59769437 |
| cg19457823 | CRHR2 | -6.5876812 | 0.00137723 | 5.58411047 | -1.179719 | 0.238112 | 0.59769437 |
| cg08929103 | CRHR1 | -2.9342153 | 0.05317243 | 2.50502233 | -1.171333 | 0.24146495 | 0.59769437 |
| cg03245912 | FK | -6.5216972 | 0.00147117 | 5.65497349 | -1.1532675 | 0.2488006 | 0.59769437 |
| cg15929276 | CRHR1 | -5.2579786 | 0.00520582 | 4.56978918 | -1.1505954 | 0.24989871 | 0.59769437 |
| cg02833950 | CRH | -6.5984235 | 0.00136251 | 5.74275485 | -1.1489997 | 0.25055611 | 0.59769437 |
| cg16755766 | GR1 | -5.4997916 | 0.00408762 | 4.93763402 | -1.1138516 | 0.26534285 | 0.59769437 |
| cg19491599 | FK | -3.4207709 | 0.03268723 | 3.09679363 | -1.104617 | 0.26932558 | 0.59769437 |
| cg18815117 | CRHR1 | 6.35098361 | 573.056095 | 5.77053496 | 1.10058836 | 0.27107585 | 0.59769437 |
| cg03667083 | GR2 | -6.385155 | 0.00168641 | 5.8016091 | -1.1005835 | 0.27107799 | 0.59769437 |
| cg19696975 | GR1 | -6.2604697 | 0.00191035 | 5.70720844 | -1.0969408 | 0.27266728 | 0.59769437 |
| cg24295963 | FK | -3.4191181 | 0.0327413 | 3.14547553 | -1.0869956 | 0.27703875 | 0.59769437 |
| cg17349736 | GR1 | -5.9611338 | 0.00257699 | 5.48917026 | -1.0859808 | 0.27748747 | 0.59769437 |
| cg13764763 | GR1 | 6.62093955 | 750.650039 | 6.14306177 | 1.07779147 | 0.28112683 | 0.59769437 |
| cg18534039 | GR1 | 7.9676557 | 2886.08359 | 7.40422357 | 1.07609605 | 0.2818843 | 0.59769437 |
| cg23430507 | CRH | -3.9695952 | 0.01888108 | 3.72027388 | -1.0670169 | 0.2859642 | 0.59769437 |
| cg14339974 | CRHR1 | -6.0845705 | 0.00227774 | 5.70566362 | -1.0664089 | 0.28623884 | 0.59769437 |
| cg02712145 | CRHR1 | -4.5536919 | 0.01052826 | 4.27731679 | -1.0646141 | 0.28705059 | 0.59769437 |
| cg27410679 | CRHR1 | 4.96693793 | 143.586542 | 4.84528824 | 1.0251068 | 0.3053128 | 0.62712899 |
| cg03546163 | GR2 | -2.8647185 | 0.05699918 | 2.85822566 | -1.0022716 | 0.31621242 | 0.64085718 |
| cg23329208 | CRHR1 | -4.8639799 | 0.0077197 | 4.89749863 | -0.9931559 | 0.32063397 | 0.64126793 |
| cg19035496 | FK | 7.7474945 | 2315.763 | 8.27737645 | 0.93598431 | 0.34928127 | 0.68683726 |
| cg01049782 | CRHR1 | -7.1701403 | 0.00076922 | 7.73794736 | -0.9266205 | 0.35412363 | 0.68683726 |
| cg14621978 | FK | -5.4203671 | 0.00442552 | 5.88437861 | -0.9211452 | 0.35697463 | 0.68683726 |
| cg27234800 | CRHR2 | -4.3951411 | 0.01233714 | 4.87424391 | -0.9017072 | 0.3672124 | 0.69770357 |
| cg19447040 | GR1 | 6.9214142 | 1013.75263 | 7.81665587 | 0.88546999 | 0.3759032 | 0.70539859 |
| cg00022871 | GR2 | -2.8164487 | 0.059818 | 3.23055672 | -0.8718153 | 0.38330915 | 0.71052428 |
| cg17509718 | FK | -6.1455635 | 0.00214297 | 7.164309 | -0.8578027 | 0.39100143 | 0.71084203 |
| cg13000004 | CRHR1 | 3.02368631 | 20.5669683 | 3.53858749 | 0.85448963 | 0.39283375 | 0.71084203 |
| cg24396090 | CRHR2 | 5.38079554 | 217.194995 | 6.38228709 | 0.84308265 | 0.39918222 | 0.71383173 |
| cg08119837 | CRH | -2.6964304 | 0.06744584 | 3.25219228 | -0.8291116 | 0.40704125 | 0.71942175 |
| cg10300814 | GR1 | 2.77699943 | 16.0707271 | 3.41603303 | 0.81293108 | 0.41625757 | 0.72264671 |
| cg11905112 | GR1 | 6.49277922 | 660.356088 | 8.06931257 | 0.80462607 | 0.4210355 | 0.72264671 |
| cg08695103 | CRHR1 | -4.1000801 | 0.01657135 | 5.1354724 | -0.7983842 | 0.42464756 | 0.72264671 |
| cg26464411 | FK | -4.1858202 | 0.01520973 | 5.39558177 | -0.7757866 | 0.43787498 | 0.72264671 |
| cg23420656 | GR1 | 5.46007217 | 235.114392 | 7.04453564 | 0.77507908 | 0.43829294 | 0.72264671 |
| cg07061368 | GR1 | 4.00295207 | 54.7595659 | 5.17553039 | 0.77343804 | 0.43926319 | 0.72264671 |
| cg00025823 | GR1 | 4.09745711 | 60.1870438 | 5.33123953 | 0.76857494 | 0.44214569 | 0.72264671 |
| cg25579735 | GR1 | -2.6544905 | 0.07033467 | 3.54873027 | -0.7480113 | 0.45445332 | 0.73486069 |
| cg20813374 | CRHR1 | -6.0005036 | 0.0024775 | 8.39377266 | -0.7148756 | 0.47468589 | 0.75949743 |
| cg00294552 | GR2 | -5.9203099 | 0.00268437 | 8.49232197 | -0.6971368 | 0.48571721 | 0.76633346 |
| cg05366813 | FK | 2.48416721 | 11.99113 | 3.66222479 | 0.67832188 | 0.49756763 | 0.76633346 |
| cg08845721 | CRHR1 | -4.2085215 | 0.01486834 | 6.20894662 | -0.6778157 | 0.49788856 | 0.76633346 |
| cg13111061 | CRHR1 | -2.819383 | 0.05964273 | 4.17150551 | -0.675867 | 0.49912508 | 0.76633346 |
| cg07733851 | GR1 | -3.1152888 | 0.04436569 | 4.82509672 | -0.6456428 | 0.51851074 | 0.78253888 |
| cg16912838 | CRHR1 | 2.87672643 | 17.7560522 | 4.47125974 | 0.64338164 | 0.51997649 | 0.78253888 |
| cg09422970 | GR1 | 3.41045583 | 30.2790433 | 5.41441116 | 0.6298849 | 0.52876989 | 0.78797082 |
| cg01972826 | GR2 | 2.74582376 | 15.5774407 | 4.43069725 | 0.61972724 | 0.53543738 | 0.78891947 |
| cg27503360 | GR2 | -3.4321499 | 0.03231739 | 5.62336222 | -0.6103377 | 0.54163814 | 0.78891947 |
| cg19920989 | GR1 | -3.3981131 | 0.0334363 | 5.61389835 | -0.6053036 | 0.54497727 | 0.78891947 |
| cg11524343 | CRHR1 | -1.301764 | 0.27205148 | 2.28362625 | -0.5700425 | 0.56864888 | 0.808794 |
| cg20753294 | GR1 | -3.0266158 | 0.04847943 | 5.42282889 | -0.5581249 | 0.57675911 | 0.808794 |
| cg16127724 | CRHR1 | -3.5931288 | 0.02751212 | 6.48032873 | -0.5544671 | 0.57925925 | 0.808794 |
| cg03423935 | GR1 | -2.1690486 | 0.1142863 | 3.91950439 | -0.5533987 | 0.57999044 | 0.808794 |
| cg20059597 | GR1 | -1.1292846 | 0.32326444 | 2.33802364 | -0.4830082 | 0.62908991 | 0.86750023 |
| cg18753072 | FK | 2.06674963 | 7.89910633 | 4.33461199 | 0.47680153 | 0.63350346 | 0.86750023 |
| cg06409316 | GR1 | 2.56078844 | 12.9460205 | 5.52538916 | 0.46345848 | 0.64303577 | 0.8726914 |
| cg24052866 | GR1 | -1.4909263 | 0.22516399 | 3.39006801 | -0.4397924 | 0.66008744 | 0.88790523 |
| cg15615793 | FK | -3.3127257 | 0.03641678 | 7.70896859 | -0.4297236 | 0.66739672 | 0.88986229 |
| cg03746860 | GR2 | -3.8438151 | 0.02141176 | 9.36078113 | -0.4106297 | 0.68134406 | 0.89532456 |
| cg09793084 | GR1 | 2.01259458 | 7.48270667 | 4.93637708 | 0.40770682 | 0.68348893 | 0.89532456 |
| cg06537391 | CRHR1 | -2.1672189 | 0.1144956 | 5.41819031 | -0.3999894 | 0.6891643 | 0.89532456 |
| cg07275757 | CRHR1 | -2.8015945 | 0.06071318 | 7.18112143 | -0.3901333 | 0.69643799 | 0.89710657 |
| cg18090898 | CRHR1 | -2.4358544 | 0.08752293 | 6.42710972 | -0.3789969 | 0.70469019 | 0.90010848 |
| cg08590638 | CRHR2 | 1.46543224 | 4.32941417 | 4.1868982 | 0.35000427 | 0.7263355 | 0.91462997 |
| cg26495008 | FK | -1.7969676 | 0.16580091 | 5.16871137 | -0.3476626 | 0.7280936 | 0.91462997 |
| cg20891006 | CRHR1 | 1.159858 | 3.18948035 | 3.88050819 | 0.29889333 | 0.76502144 | 0.95117691 |
| cg10556198 | CRHR1 | -1.8771085 | 0.15303196 | 6.56466171 | -0.2859414 | 0.77492301 | 0.95117691 |
| cg12969488 | GR1 | -2.1137446 | 0.12078482 | 7.42739542 | -0.2845876 | 0.77596011 | 0.95117691 |
| cg20329958 | FK | -1.5278122 | 0.21700992 | 5.85042068 | -0.2611457 | 0.79398015 | 0.96143115 |
| cg08423118 | CRHR1 | -0.6608617 | 0.51640614 | 2.56881848 | -0.2572629 | 0.79697582 | 0.96143115 |
| cg25114611 | CRHR2 | 0.95341998 | 2.59456786 | 4.14081918 | 0.23024912 | 0.81789819 | 0.97560359 |
| cg07335874 | CRHR1 | -0.9284343 | 0.39517194 | 4.35549732 | -0.2131638 | 0.83119921 | 0.97560359 |
| cg26035844 | CRH | -0.8172518 | 0.44164371 | 3.87054215 | -0.2111466 | 0.83277288 | 0.97560359 |
| cg17061760 | CRHR1 | -0.651077 | 0.52148384 | 3.31709406 | -0.1962793 | 0.84439154 | 0.97560359 |
| cg15117716 | CRHR1 | 0.35963297 | 1.43280343 | 1.9899189 | 0.18072745 | 0.85658151 | 0.97560359 |
| cg21701890 | CRH | -1.4183295 | 0.24211814 | 7.97862297 | -0.1777662 | 0.85890659 | 0.97560359 |
| cg01961214 | CRHR1 | -0.9825633 | 0.37435028 | 6.16106189 | -0.1594795 | 0.87329107 | 0.97560359 |
| cg27122725 | FK | -0.9820441 | 0.3745447 | 6.31181839 | -0.1555881 | 0.87635767 | 0.97560359 |
| cg07742588 | GR1 | 1.27341246 | 3.57302457 | 8.38971392 | 0.15178258 | 0.87935842 | 0.97560359 |
| cg02665568 | CRHR1 | 0.54422117 | 1.72326574 | 3.66632028 | 0.14843798 | 0.88199713 | 0.97560359 |
| cg09143276 | CRHR2 | -1.1247549 | 0.32473206 | 7.77321731 | -0.1446962 | 0.88495075 | 0.97560359 |
| cg07715663 | CRHR1 | 0.80926836 | 2.24626392 | 5.63204831 | 0.14368988 | 0.88574536 | 0.97560359 |
| cg01913022 | FK | -1.0383047 | 0.3540544 | 8.73891563 | -0.1188139 | 0.90542279 | 0.98284314 |
| cg12512584 | GR2 | 0.74013621 | 2.09622103 | 6.4595468 | 0.11458021 | 0.90877787 | 0.98284314 |
| cg01448078 | FK | 0.56678176 | 1.76258549 | 5.16527985 | 0.10972915 | 0.91262418 | 0.98284314 |
| cg14642437 | CRHR1 | 0.47349115 | 1.60558978 | 5.56372472 | 0.08510327 | 0.93217929 | 0.98284314 |
| cg14297797 | GR2 | 0.06179446 | 1.06374368 | 0.82794017 | 0.07463638 | 0.94050403 | 0.98284314 |
| cg13947929 | FK | 0.2564259 | 1.29230301 | 3.46826855 | 0.07393485 | 0.94106223 | 0.98284314 |
| cg12946179 | CRHR2 | 0.41875582 | 1.52006913 | 5.96574282 | 0.07019341 | 0.94403972 | 0.98284314 |
| cg05039098 | FK | -0.2741839 | 0.76019229 | 4.20375842 | -0.0652235 | 0.94799605 | 0.98284314 |
| cg16642545 | CRHR2 | -0.2480575 | 0.78031508 | 3.99687523 | -0.0620629 | 0.95051278 | 0.98284314 |
| cg03405789 | GR1 | -0.1799905 | 0.83527815 | 3.96151979 | -0.0454347 | 0.96376082 | 0.98880668 |
| cg00130530 | CRHR1 | 0.18408713 | 1.20212056 | 5.69734936 | 0.03231101 | 0.97422403 | 0.98880668 |
| cg16052510 | GR2 | 0.27105349 | 1.31134522 | 8.93392751 | 0.03033979 | 0.97579606 | 0.98880668 |
| cg25563198 | GR2 | 0.05243975 | 1.05383907 | 7.42771868 | 0.00706001 | 0.99436698 | 0.99558127 |
| cg18718518 | GR1 | 0.01706714 | 1.01721362 | 3.08177625 | 0.00553809 | 0.99558127 | 0.99558127 |

Note. The coefficients in cox survival models are interpreted the same as regression coefficients, and the exponents gives you the hazard for developing depression.

Supplemental Table 3. All tested models after adding SNP PCs

| CpG | Gene | coef | exp | se | z | PVal | Adj.P |
| --- | --- | --- | --- | --- | --- | --- | --- |
| cg27605489 | CRHR2 | -24.936206 | 1.48E-11 | 6.73524816 | -3.702344 | 0.00021362 | 0.03246974 |
| cg24801588 | GR1 | -21.007212 | 7.53E-10 | 6.50593735 | -3.2289293 | 0.00124255 | 0.09443349 |
| cg00407401 | GR1 | -15.31712 | 2.23E-07 | 5.04210923 | -3.0378398 | 0.00238281 | 0.10617797 |
| cg05900547 | GR1 | -7.0387989 | 0.00087718 | 2.35449093 | -2.9895205 | 0.00279416 | 0.10617797 |
| cg07508782 | CRHR1 | 10.1536038 | 25683.4932 | 3.68092647 | 2.758437 | 0.00580785 | 0.13253678 |
| cg03238273 | CRHR1 | -13.360037 | 1.58E-06 | 4.92213239 | -2.7142783 | 0.00664204 | 0.13253678 |
| cg16535116 | GR1 | -9.2055004 | 0.00010049 | 3.43471867 | -2.6801323 | 0.00735931 | 0.13253678 |
| cg23409074 | CRH | -8.2953215 | 0.00024968 | 3.11861018 | -2.6599418 | 0.00781542 | 0.13253678 |
| cg17924854 | CRHR2 | -14.486627 | 5.11E-07 | 5.44905349 | -2.6585585 | 0.00784757 | 0.13253678 |
| cg05877083 | CRHR2 | -24.805331 | 1.69E-11 | 9.62963881 | -2.5759358 | 0.00999692 | 0.15195319 |
| cg05087823 | CRHR1 | -13.151516 | 1.94E-06 | 5.60275301 | -2.347331 | 0.01890845 | 0.26128035 |
| cg06952416 | GR1 | -17.017359 | 4.07E-08 | 7.57253836 | -2.2472464 | 0.02462429 | 0.31190761 |
| cg06087101 | FKBP5 | 5.34763606 | 210.11102 | 2.54789561 | 2.09884425 | 0.03583063 | 0.35761596 |
| cg08215831 | CRH | -11.285701 | 1.26E-05 | 5.42091513 | -2.0818811 | 0.03735333 | 0.35761596 |
| cg01972879 | CRHR2 | -13.15705 | 1.93E-06 | 6.32719772 | -2.0794435 | 0.03757661 | 0.35761596 |
| cg24353392 | CRHR1 | -12.449441 | 3.92E-06 | 6.03858981 | -2.0616471 | 0.03924134 | 0.35761596 |
| cg18640030 | CRH | -6.6950912 | 0.00123697 | 3.25987958 | -2.0537848 | 0.03999652 | 0.35761596 |
| cg25719092 | CRHR1 | -7.7615313 | 0.0004258 | 3.83721102 | -2.0227012 | 0.04310396 | 0.36328889 |
| cg04227637 | CRH | -11.703822 | 8.26E-06 | 5.85987076 | -1.9972833 | 0.04579442 | 0.36328889 |
| cg22046703 | CRHR1 | -14.650106 | 4.34E-07 | 7.40228945 | -1.9791318 | 0.04780117 | 0.36328889 |
| cg27225476 | GR2 | -9.2747779 | 9.38E-05 | 4.9573071 | -1.8709307 | 0.06135469 | 0.43293304 |
| cg19650300 | GR2 | -12.062195 | 5.77E-06 | 6.47952346 | -1.8615868 | 0.06266136 | 0.43293304 |
| cg03591753 | FKBP5 | -9.2041779 | 0.00010062 | 5.01827484 | -1.8341319 | 0.06663442 | 0.4375716 |
| cg06843189 | GR2 | -5.483113 | 0.00415637 | 3.06745503 | -1.7875121 | 0.07385476 | 0.4375716 |
| cg04856689 | CRHR1 | -11.826816 | 7.31E-06 | 6.68985991 | -1.767872 | 0.07708231 | 0.4375716 |
| cg05437692 | GR2 | 10.0363788 | 22842.5152 | 5.74240579 | 1.74776551 | 0.08050464 | 0.4375716 |
| cg09238384 | GR2 | -8.1958615 | 0.00027579 | 4.68946526 | -1.7477177 | 0.08051292 | 0.4375716 |
| cg23416081 | FKBP5 | -5.9606854 | 0.00257814 | 3.41159419 | -1.7471848 | 0.0806053 | 0.4375716 |
| cg01294526 | GR1 | 12.3513845 | 231280.028 | 7.31107129 | 1.6894083 | 0.09114121 | 0.47770566 |
| cg18998365 | GR1 | -7.3277022 | 0.00065708 | 4.38532126 | -1.6709613 | 0.09472932 | 0.47996187 |
| cg03857453 | GR1 | -7.483767 | 0.00056214 | 4.52208744 | -1.6549364 | 0.09793741 | 0.48020922 |
| cg06475362 | CRHR1 | -7.0266887 | 0.00088787 | 4.44985126 | -1.579084 | 0.1143168 | 0.51272317 |
| cg03066966 | CRHR1 | -13.101089 | 2.04E-06 | 8.41897004 | -1.5561392 | 0.11967499 | 0.51272317 |
| cg06175988 | CRHR2 | -7.3933275 | 0.00061535 | 4.77007732 | -1.5499387 | 0.12115623 | 0.51272317 |
| cg16664570 | CRH | -10.176801 | 3.80E-05 | 6.60529468 | -1.5407035 | 0.12338895 | 0.51272317 |
| cg10993059 | GR2 | -6.392858 | 0.00167347 | 4.15915609 | -1.5370565 | 0.12427947 | 0.51272317 |
| cg19014730 | FKBP5 | -7.7971298 | 0.00041091 | 5.07988385 | -1.5349032 | 0.12480761 | 0.51272317 |
| cg17342132 | GR1 | 8.38887457 | 4397.8654 | 5.52506335 | 1.51833093 | 0.12893099 | 0.51572398 |
| cg20598211 | GR1 | 11.5872496 | 107715.588 | 7.87056061 | 1.47222672 | 0.14095967 | 0.53328171 |
| cg20728768 | GR1 | 7.80019579 | 2441.07987 | 5.39161836 | 1.44672625 | 0.14797361 | 0.53328171 |
| cg25708981 | GR1 | -5.1358581 | 0.005882 | 3.5627654 | -1.441537 | 0.14943304 | 0.53328171 |
| cg24063856 | CRHR1 | -7.0963104 | 0.00082816 | 4.93354183 | -1.4383805 | 0.15032612 | 0.53328171 |
| cg19226017 | FKBP5 | -6.8973733 | 0.00101044 | 4.93691487 | -1.3971019 | 0.16238292 | 0.53328171 |
| cg21789597 | FKBP5 | -8.0704605 | 0.00031264 | 5.8194572 | -1.3868064 | 0.1655008 | 0.53328171 |
| cg03906910 | GR1 | -3.3555899 | 0.03488878 | 2.4416069 | -1.3743367 | 0.16933718 | 0.53328171 |
| cg17578833 | CRH | -5.6839653 | 0.00340005 | 4.14694146 | -1.3706403 | 0.1704871 | 0.53328171 |
| cg16219186 | GR1 | 7.333937 | 1531.39904 | 5.39849536 | 1.35851502 | 0.17430032 | 0.53328171 |
| cg11760414 | CRHR1 | -6.2521279 | 0.00192635 | 4.62702814 | -1.3512189 | 0.17662532 | 0.53328171 |
| cg23776787 | GR1 | -4.5107776 | 0.01098991 | 3.36605544 | -1.3400782 | 0.18021991 | 0.53328171 |
| cg21517946 | FKBP5 | 2.9303543 | 18.7342669 | 2.20103799 | 1.33135108 | 0.18307352 | 0.53328171 |
| cg07817266 | CRHR1 | 6.00992959 | 407.454632 | 4.53604141 | 1.32492829 | 0.18519494 | 0.53328171 |
| cg19645279 | GR1 | 7.57206662 | 1943.15188 | 5.72357617 | 1.32296075 | 0.18584843 | 0.53328171 |
| cg06613263 | GR1 | 7.39862776 | 1633.74101 | 5.602177 | 1.32067012 | 0.18661138 | 0.53328171 |
| cg18484679 | GR1 | -4.90344 | 0.00742101 | 3.75501484 | -1.3058377 | 0.19160775 | 0.53328171 |
| cg01819552 | CRHR2 | -8.0645042 | 0.00031451 | 6.24614372 | -1.2911173 | 0.19666301 | 0.53328171 |
| cg12888360 | GR1 | -6.6691793 | 0.00126944 | 5.19882443 | -1.2828245 | 0.19955357 | 0.53328171 |
| cg19457823 | GR1 | 5.02246984 | 151.785727 | 3.96384103 | 1.26707146 | 0.20512974 | 0.53328171 |
| cg08929103 | CRHR1 | -9.6620467 | 6.37E-05 | 7.64698045 | -1.2635114 | 0.20640547 | 0.53328171 |
| cg03245912 | FKBP5 | -6.9943831 | 0.00091702 | 5.54289502 | -1.2618646 | 0.2069975 | 0.53328171 |
| cg15929276 | FKBP5 | -4.1172659 | 0.01628899 | 3.44032848 | -1.1967654 | 0.23139802 | 0.56857992 |
| cg02833950 | CRHR1 | -2.9123286 | 0.05434902 | 2.49447419 | -1.167512 | 0.24300365 | 0.56857992 |
| cg16755766 | CRHR2 | -6.4645071 | 0.00155776 | 5.54371818 | -1.1660959 | 0.24357567 | 0.56857992 |
| cg19491599 | GR2 | -3.3736916 | 0.03426292 | 2.90062928 | -1.1630895 | 0.24479318 | 0.56857992 |
| cg18815117 | CRHR1 | 5.20753101 | 182.642559 | 4.53338805 | 1.14870621 | 0.25067714 | 0.56857992 |
| cg03667083 | CRHR2 | -5.593011 | 0.0037238 | 4.9403951 | -1.1320979 | 0.25759327 | 0.56857992 |
| cg19696975 | CRH | -4.172907 | 0.01540741 | 3.68916388 | -1.1311254 | 0.25800231 | 0.56857992 |
| cg24295963 | FKBP5 | -6.402572 | 0.00165729 | 5.67798055 | -1.1276143 | 0.25948285 | 0.56857992 |
| cg17349736 | GR1 | -6.5601818 | 0.00141563 | 5.83664692 | -1.1239641 | 0.26102824 | 0.56857992 |
| cg13764763 | GR1 | 9.26285506 | 10539.1805 | 8.26552222 | 1.12066181 | 0.26243185 | 0.56857992 |
| cg18534039 | CRHR1 | -6.5326177 | 0.00145519 | 5.84105564 | -1.1183968 | 0.26339758 | 0.56857992 |
| cg23430507 | GR1 | 7.2527527 | 1411.98628 | 6.51473987 | 1.11328355 | 0.26558667 | 0.56857992 |
| cg14339974 | FKBP5 | -3.3834464 | 0.03393032 | 3.08691438 | -1.096061 | 0.27305209 | 0.57066222 |
| cg02712145 | CRHR2 | 9.92261567 | 20386.2446 | 9.0721701 | 1.09374224 | 0.27406804 | 0.57066222 |
| cg27410679 | CRHR1 | 6.34010483 | 566.855734 | 5.8665013 | 1.08073015 | 0.27981717 | 0.57215556 |
| cg03546163 | FKBP5 | -3.3471334 | 0.03518507 | 3.1300564 | -1.0693524 | 0.28491089 | 0.57215556 |
| cg23329208 | GR2 | -5.9358633 | 0.00264294 | 5.58537989 | -1.0627502 | 0.28789527 | 0.57215556 |
| cg19035496 | CRH | -6.1338229 | 0.00216828 | 5.79499874 | -1.0584684 | 0.28984196 | 0.57215556 |
| cg01049782 | CRHR2 | 4.13294515 | 62.3613161 | 4.0238528 | 1.02711142 | 0.304368 | 0.58391381 |
| cg14621978 | GR1 | 3.99283493 | 54.2083484 | 3.89666912 | 1.02467898 | 0.30551469 | 0.58391381 |
| cg27234800 | GR2 | -5.393342 | 0.00454675 | 5.2831608 | -1.0208552 | 0.30732306 | 0.58391381 |
| cg19447040 | CRHR1 | 4.77804167 | 118.871333 | 4.883549 | 0.97839536 | 0.32787882 | 0.6094409 |
| cg00022871 | CRHR1 | -8.8176653 | 0.00014809 | 9.06581932 | -0.9726275 | 0.33073847 | 0.6094409 |
| cg17509718 | CRHR1 | -4.7882086 | 0.00832736 | 4.94533208 | -0.9682279 | 0.33293055 | 0.6094409 |
| cg13000004 | GR2 | -3.4215552 | 0.0326616 | 3.61299415 | -0.9470138 | 0.34363176 | 0.6094409 |
| cg24396090 | GR2 | -8.7513602 | 0.00015825 | 9.33737945 | -0.9372394 | 0.34863541 | 0.6094409 |
| cg08119837 | CRHR1 | 3.56955261 | 35.5007069 | 3.83083217 | 0.93179561 | 0.35144216 | 0.6094409 |
| cg10300814 | FKBP5 | 7.64162986 | 2083.13627 | 8.30748525 | 0.91984874 | 0.35765181 | 0.6094409 |
| cg11905112 | FKBP5 | -5.408496 | 0.00447837 | 5.88218918 | -0.9194699 | 0.35784983 | 0.6094409 |
| cg08695103 | GR1 | -5.0799547 | 0.00622019 | 5.55882875 | -0.9138534 | 0.36079387 | 0.6094409 |
| cg26464411 | GR1 | 7.50759188 | 1821.82108 | 8.21632728 | 0.91374061 | 0.36085316 | 0.6094409 |
| cg23420656 | CRHR1 | -6.8483237 | 0.00106123 | 7.79249334 | -0.878836 | 0.3794902 | 0.63387375 |
| cg07061368 | FKBP5 | -6.056725 | 0.00234206 | 7.40481588 | -0.817944 | 0.41338914 | 0.68299076 |
| cg00025823 | CRHR1 | -5.1032154 | 0.00607718 | 6.4820945 | -0.7872788 | 0.4311187 | 0.69340458 |
| cg25579735 | GR1 | -2.9014705 | 0.05494237 | 3.74935265 | -0.7738591 | 0.43901414 | 0.69340458 |
| cg20813374 | FKBP5 | -4.18365 | 0.01524277 | 5.40735704 | -0.7736959 | 0.43911064 | 0.69340458 |
| cg00294552 | GR1 | -2.2501149 | 0.10538712 | 2.94259849 | -0.7646694 | 0.44446846 | 0.69340458 |
| cg05366813 | CRHR2 | 4.66715745 | 106.39488 | 6.11245746 | 0.76354846 | 0.44513638 | 0.69340458 |
| cg08845721 | GR1 | 4.12507134 | 61.872223 | 5.46971493 | 0.75416569 | 0.45074974 | 0.69340458 |
| cg13111061 | CRHR1 | -4.2288371 | 0.01456932 | 5.61816357 | -0.7527081 | 0.45162535 | 0.69340458 |
| cg07733851 | GR1 | -3.6148965 | 0.02691971 | 5.05137547 | -0.7156262 | 0.47422221 | 0.7198751 |
| cg16912838 | FKBP5 | 2.60259511 | 13.4987234 | 3.6709104 | 0.70897811 | 0.47833806 | 0.7198751 |
| cg09422970 | CRHR1 | 3.08378008 | 21.8408065 | 4.47194372 | 0.68958383 | 0.49045594 | 0.73087552 |
| cg01972826 | CRH | -2.1731812 | 0.11381497 | 3.27664155 | -0.6632343 | 0.50718048 | 0.7451922 |
| cg27503360 | CRHR1 | -5.3181928 | 0.0049016 | 8.07515448 | -0.6585871 | 0.51016093 | 0.7451922 |
| cg19920989 | CRHR2 | -5.2839528 | 0.00507234 | 8.11135129 | -0.6514269 | 0.51477092 | 0.7451922 |
| cg11524343 | CRHR1 | 1.56515328 | 4.78340809 | 2.71516669 | 0.57644832 | 0.56431219 | 0.8080677 |
| cg20753294 | GR1 | -3.0792768 | 0.04599251 | 5.5150389 | -0.5583418 | 0.57661101 | 0.8080677 |
| cg16127724 | CRHR2 | 3.37071295 | 29.099266 | 6.11096202 | 0.55158467 | 0.58123294 | 0.8080677 |
| cg03423935 | CRHR1 | -3.6352821 | 0.02637649 | 6.67964067 | -0.5442332 | 0.58628102 | 0.8080677 |
| cg20059597 | CRHR1 | 2.42469596 | 11.2987937 | 4.50179931 | 0.53860597 | 0.59015877 | 0.8080677 |
| cg18753072 | CRHR1 | -2.7539567 | 0.06367542 | 5.14101968 | -0.535683 | 0.59217767 | 0.8080677 |
| cg06409316 | FKBP5 | 2.35601542 | 10.548835 | 4.43693482 | 0.53100068 | 0.59541831 | 0.8080677 |
| cg24052866 | GR1 | 2.67955791 | 14.5786467 | 5.17874691 | 0.51741434 | 0.60486695 | 0.81362634 |
| cg15615793 | CRHR2 | 2.10517307 | 8.20852349 | 4.44768755 | 0.47331856 | 0.63598592 | 0.83452413 |
| cg03746860 | GR1 | -3.2164265 | 0.0400981 | 6.80516964 | -0.4726446 | 0.63646678 | 0.83452413 |
| cg09793084 | CRHR1 | -1.3118544 | 0.26932016 | 2.77891452 | -0.4720744 | 0.63687368 | 0.83452413 |
| cg06537391 | CRHR1 | -1.1013186 | 0.33243245 | 2.45980266 | -0.4477264 | 0.65435066 | 0.84451348 |
| cg07275757 | GR2 | -3.8854012 | 0.02053959 | 8.71198363 | -0.4459835 | 0.65560915 | 0.84451348 |
| cg18090898 | CRHR2 | 1.81961517 | 6.16948382 | 4.28841968 | 0.42430903 | 0.67134046 | 0.8575105 |
| cg08590638 | CRHR1 | -2.5225523 | 0.08025452 | 6.45611882 | -0.3907227 | 0.69600221 | 0.87881656 |
| cg26495008 | FKBP5 | -3.00937 | 0.04932274 | 7.79868403 | -0.3858818 | 0.69958423 | 0.87881656 |
| cg20891006 | CRHR1 | -2.7035881 | 0.06696481 | 7.19353107 | -0.375836 | 0.70703881 | 0.88090081 |
| cg10556198 | CRHR1 | -1.9788329 | 0.13823048 | 5.45681265 | -0.3626353 | 0.71687734 | 0.88285366 |
| cg12969488 | GR1 | 1.85484888 | 6.3907324 | 5.18576055 | 0.35768117 | 0.72058193 | 0.88285366 |
| cg20329958 | CRH | -2.8835507 | 0.0559358 | 8.22907477 | -0.3504101 | 0.72603097 | 0.88285366 |
| cg08423118 | GR1 | -1.2479389 | 0.28709592 | 3.74753613 | -0.3330025 | 0.73913239 | 0.88498081 |
| cg25114611 | FKBP5 | -1.7217196 | 0.17875848 | 5.17631045 | -0.3326152 | 0.73942475 | 0.88498081 |
| cg07335874 | GR2 | 1.47737143 | 4.38141369 | 4.5708679 | 0.32321464 | 0.74653269 | 0.88650757 |
| cg26035844 | GR2 | 1.93451884 | 6.92071331 | 6.64481257 | 0.29113219 | 0.77095022 | 0.90840646 |
| cg17061760 | GR2 | 0.22568301 | 1.25317836 | 0.82725378 | 0.27280989 | 0.78499936 | 0.91278202 |
| cg15117716 | CRHR1 | -0.9147669 | 0.40061 | 3.47524418 | -0.2632238 | 0.79237811 | 0.91278202 |
| cg21701890 | GR2 | 1.96040088 | 7.10217359 | 7.45872541 | 0.26283323 | 0.79267912 | 0.91278202 |
| cg01961214 | CRHR1 | -1.0122618 | 0.36339612 | 4.43473515 | -0.2282576 | 0.81944602 | 0.91446947 |
| cg27122725 | GR1 | -2.0847113 | 0.12434301 | 9.22456884 | -0.2259955 | 0.8212049 | 0.91446947 |
| cg07742588 | GR1 | -0.6568916 | 0.51846043 | 2.92666306 | -0.2244507 | 0.82240662 | 0.91446947 |
| cg02665568 | FKBP5 | -1.2888131 | 0.27559771 | 5.9371284 | -0.2170768 | 0.82814847 | 0.91446947 |
| cg09143276 | GR2 | -1.4449125 | 0.2357667 | 6.72332361 | -0.2149105 | 0.82983713 | 0.91446947 |
| cg07715663 | GR1 | -1.3055177 | 0.2710322 | 6.18000819 | -0.2112485 | 0.83269334 | 0.91446947 |
| cg01913022 | CRHR2 | 0.83839036 | 2.31264145 | 4.05642778 | 0.20668194 | 0.83625826 | 0.91446947 |
| cg12512584 | CRHR1 | -0.8787005 | 0.41532228 | 6.19441484 | -0.1418537 | 0.88719559 | 0.96100271 |
| cg01448078 | CRHR1 | 0.74554258 | 2.10758467 | 5.66677041 | 0.13156393 | 0.89532922 | 0.96100271 |
| cg14642437 | FKBP5 | -1.121439 | 0.3258106 | 8.73846304 | -0.1283337 | 0.89788492 | 0.96100271 |
| cg14297797 | CRHR1 | 0.44646926 | 1.56278464 | 3.8093572 | 0.11720331 | 0.90669895 | 0.96100271 |
| cg13947929 | CRHR1 | -0.6184271 | 0.53879124 | 6.10001114 | -0.1013813 | 0.91924778 | 0.96100271 |
| cg12946179 | GR2 | -0.9075986 | 0.40349201 | 9.05407753 | -0.100242 | 0.92015223 | 0.96100271 |
| cg05039098 | FKBP5 | -0.6247146 | 0.53541424 | 6.46907618 | -0.0965694 | 0.92306839 | 0.96100271 |
| cg16642545 | CRHR1 | -0.5535591 | 0.57490005 | 6.31298473 | -0.0876858 | 0.93012641 | 0.96176336 |
| cg03405789 | CRH | 0.25438894 | 1.28967332 | 3.92350526 | 0.06483716 | 0.94830365 | 0.96778897 |
| cg00130530 | FKBP5 | 0.26900433 | 1.3086608 | 5.31738344 | 0.05058961 | 0.95965255 | 0.96778897 |
| cg16052510 | FKBP5 | 0.17194865 | 1.18761685 | 3.4925408 | 0.04923311 | 0.96073352 | 0.96778897 |
| cg25563198 | FKBP5 | -0.1924059 | 0.82497197 | 4.20781818 | -0.0457258 | 0.9635288 | 0.96778897 |
| cg18718518 | GR1 | -0.2111926 | 0.80961814 | 5.22993262 | -0.0403815 | 0.96778897 | 0.96778897 |

Supplemental Figures 1a-f. Heatmaps of correlations among CpG beta values and genotypes within each candidate gene.

a.

b.

c.

d.

e.

f.
